# Identification of optimum scopes of environmental factors for snails using spatial analysis techniques in Dongting Lake Region, China

**DOI:** 10.1186/1756-3305-7-216

**Published:** 2014-05-09

**Authors:** Jin-Yi Wu, Yi-Biao Zhou, Lin-Han Li, Sheng-Bang Zheng, Song Liang, Ashley Coatsworth, Guang-Hui Ren, Xiu-Xia Song, Zhong He, Bin Cai, Jia-Bian You, Qing-Wu Jiang

**Affiliations:** 1 Department of Epidemiology, School of Public Health, Fudan University, 138 Yi Xue Yuan Road, Shanghai 200032, China; 2 Key Laboratory of Public Health Safety, Ministry of Education, Fudan University, 138 Yi Xue Yuan Road, Shanghai 200032, China; 3 Center for Tropical Disease Research, Fudan University, 138 Yi Xue Yuan Road, Shanghai 200032, China; 4 Department of Environmental and Global Health, College of Public Health and Health Professions, University of Florida, Gainesville, FL, USA; 5 Emerging Pathogens Institute, University of Florida, Gainesville, FL, USA; 6 Department of Epidemiology, College of Public Health and Health Professions, University of Florida, Gainesville, FL, USA; 7 Hunan station for Schistosomiasis Control, Changsha, Hunan Province 410000, China; 8 Junshan office of Leading Group for Schistosomiasis Control, Yueyang, Hunan province 414000, China; 9 Junshan station for Schistosomiasis Control, Yueyang, Hunan Province 414000, China; 10 Qianlianghu station for Schistosomiasis Control, Yueyang, Hunan Province 414000, China

**Keywords:** *Schistosomiasis japonica*, *Oncomelania hupensis*, Environmental factors, Spatial clustering, GWR

## Abstract

**Background:**

Owing to the harmfulness and seriousness of *Schistosomiasis japonica* in China, the control and prevention of *S. japonica* transmission are imperative. As the unique intermediate host of this disease, *Oncomelania hupensis* plays an important role in the transmission. It has been reported that the snail population in Qiangliang Lake district, Dongting Lake Region has been naturally declining and is slowly becoming extinct. Considering the changes of environmental factors that may cause this phenomenon, we try to explore the relationship between circumstance elements and snails, and then search for the possible optimum scopes of environmental factors for snails.

**Methods:**

Moisture content of soil, pH, temperature of soil and elevation were collected by corresponding apparatus in the study sites. The LISA statistic and GWR model were used to analyze the association between factors and mean snail density, and the values in high-high clustered areas and low-low clustered areas were extracted to find out the possible optimum ranges of these elements for snails.

**Results:**

A total of 8,589 snail specimens were collected from 397 sampling sites in the study field. Besides the mean snail density, three environmental factors including water content, pH and temperature had high spatial autocorrelation. The spatial clustering suggested that the possible optimum scopes of moisture content, pH, temperature of the soil and elevation were 58.70 to 68.93%, 6.80 to 7.80, 22.73 to 24.23°C and 23.50 to 25.97 m, respectively. Moreover, the GWR model showed that the possible optimum ranges of these four factors were 36.58 to 61.08%, 6.541 to 6.89, 24.30 to 25.70°C and 23.50 to 29.44 m, respectively.

**Conclusion:**

The results indicated the association between snails and environmental factors was not linear but U-shaped. Considering the results of two analysis methods, the possible optimum scopes of moisture content, pH, temperature of the soil and elevation were 58.70% to 68.93%, 6.6 to 7.0, 22.73°C to 24.23°C, and 23.5 m to 26.0 m, respectively. The findings in this research will help in making an effective strategy to control snails and provide a method to analyze other factors.

## Background

Schistosomiasis, a snail-borne parasitic disease of public health, leads to chronic ill-health, with poverty exacerbating its negative health effects. It affects almost 240 million people worldwide, and more than 700 million people live in endemic areas [1]. *Schistosomiasis japonica* is the most hazardous disease type of the five kinds of Schistosomiasis, and it is difficult to prevent and treat [2,3]. *S. japonica* has existed in China for over 2000 years, and 671.3 thousand people were still infected with *S. japonica* until 2006 in seven provinces [4-7].

*Oncomelania hupensis*, found mostly in marshland and lake areas, is the sole intermediate host of *S. japonicum*. It is closely associated with the transmission and epidemic of *S.japonica*. The distribution of snails is consistent with the epidemic area of *S. japonica*[8,9]. The Three Gorges Dam (TGD) is one of several tremendous engineering projects transforming China’s ecology and environmental circumstance. However, the construction of Three Gorges Dam (TGD) and the implementation of the South-to-North Water Diversion Project (SNWDP) were reported to influence the surrounding ecological environment, which might affect the distribution of snails [10-12]. Interestingly, *Oncomelania hupensis* has declined naturally in Qiangliang Lake district, Dongting Lake Region since 1990, and snails have not been found in the region since 2000 [13]. The Qiangliang Lake district is located in the northwestern Dongting Lake. The area of this region where snails used to live and breed is about 433.2 km^2^. Therefore, the question of why and how this phenomenon of natural population decline has occurred is undetermined. The possible optimum scopes of environmental factors for the snails captures our interest, which will help explain what drives this natural population decline.

With the development of spatial techniques, increasingly more public health problems have been analyzed using spatial modeling [12,14-24]. In previous research, experiments and field trials have both been used. Prior analyses of the relationship between snails and environmental factors commonly adopted a global model (i.e. ordinary least square regression model (OLS)), which only offered reliable information without considering the spatial variability. Geographically weighted regression (GWR) model overcame this problem, as it made use of spatial information adequately [18,25-28]. GWR is a new local modeling technique for conducting spatial analysis. This technique allows local as opposed to global models of relationships to be measured and mapped. The function is improved with spatial matrix on the basis of the OLS model. Besides GWR, Local Indicators of Spatial Association (LISA) was also used to analyze the data. LISA allows for the decomposition of global indicators, such as Moran’s I, into the contribution of each individual observation. The LISA for each observation gives an indication of the extent of significant spatial clustering of similar values around that observation, and the sum of LISAs for all observations is proportional to a global indicator of spatial association [29]. We utilized these techniques to extract useful information and to identify the possible suitable ranges for snails. This study aimed to explore the possible optimum scopes of environmental factors for snails using spatial analysis techniques (both GWR and LISA) in their natural habitats.

## Methods

### Study area

**Figure 1:**
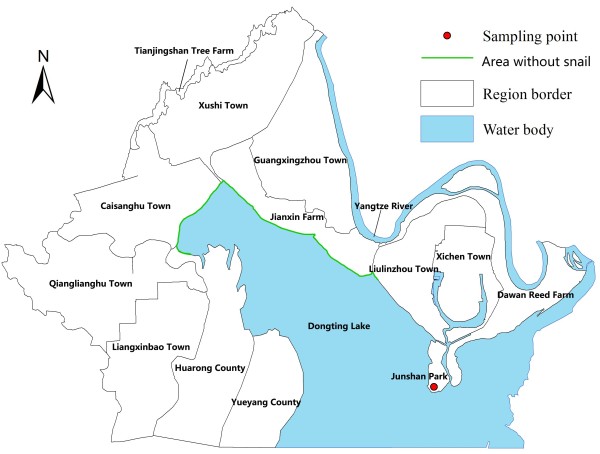
**Location of sampled bottomland in Dongting Lake Region.** Detailed legends: This map shows where the sampled bottomland is located in.

The study was conducted in a bottomland of Dongting Lake Region. Dongting Lake is located at 28°30′–30°20′N and 111°40′–113°40′E in the northeastern part of Hunan Province and covers a water surface area of 2,681 km^2^. Dongting Lake is the second largest water source in China and plays an important role in regulating water levels in the Yangtze River. *S. japonica* has been endemic in the Dongting Lake region for centuries, and it has had a devastating effect on the public health of the local people [30-36]. A bottomland with 16 km^2^, which is adjacent to Qiangliang Lake district, was selected as our study field (Figure 1). The bottomland of Dongting Lake Region has an obvious characteristic that water arises in summer and land appears in winter. Rainfall pattern in the area is seasonal, with the heaviest rain falling from April to June and the lightest rain dropping from December to February. This kind of environment is quite suitable for snails to live and reproduce, meaning that Dongting Lake Region has provided optimal circumstances for *S. japonica* for a long time [37-41].

### Snail sampling

Snail sampling was conducted from May 6th to May 10th in 2013 to obtain samples of snails in the bottomland of Dongting Lake Region. This bottomland area in Dongting Lake Region has a typical environment suitable for snail survival. Adopting systematic sampling methods, snail sampling was executed by 10 well-trained collectors working in the local station for schistosomiasis control for four days. We used tweezers and paper bags to collect the snails on the surface of bottomland. The horizontal and vertical distance between sampling points were both 20 m, and the sampling area per point was about 0.11 m^2^. We selected 50 points in the horizontal direction, while also selecting 10 lines in the vertical direction. The total area of sampling site is 10,000 m^2^. In each collection, gathered snails were appropriately labeled and transported to the laboratory of the local station for schistosomiasis control.

After collection, snails were transported in flasks containing 5 ml of clear, filtered water. After four hours, the numbers of dead and alive snails were counted. The water in the flasks was used to make smears to examine for the existence of cercariae under a microscope. Snails were killed using niclosamide.

### Environmental factor sampling

Sampling of environmental factors simultaneously coincided with snail sampling. While measuring environmental factors, the weather generally remained consistent, providing mostly sunny weather. Four elements including water content, pH, elevation and temperature were collected using professional equipment containing a GPS handheld PC, a moisture meter, and a pH meter (which can detect both pH and temperature). All these factors were gathered in the snail sampling points. Once recording was complete, these parameters were checked, and then matched with mean snail density.

Water content of soil was measured using a Soil Moisture Meter (SieldScout TDR 300, Spectrum Ltd, USA). The accuracy of this instrument was ±3.0% volumetric water content. The probe of the moisture meter was put into soil 15 cm under the surface to detect the water content, and the data was recorded when the water content reading was steady. After the process of collecting the data, all the information would be exported into a computer. To make the data accurate, adjustment for soil moisture was needed. Thirty sampling points were randomly selected from the snail sampling points, to collect samples of soil weighing 30 g in 15 cm deep for soil moisture assessment. Soil samples were placed into a plastic container to prevent the change of properties in soil. The water content of these sampling soils was tested using the drying method [42]. Finally, a calibration curve was calculated, and all the information of water moisture corrected accordingly.

Temperature and pH of soil were measured with Portable Waterproof pH/ORP/C Meter (HANNA HI991002N, Hanna Instruments Ltd, Italy). The accuracy of this equipment was ±1°C outside and ±0.02 pH. The glass probe of the meter was put into soil 15 cm under the surface, and the data was recorded when the reading was steady. Before the next measurement, the glass probe was washed by distilled water. To obtain the pH value, we collected the 15 cm deep soil weighing 30 g (the number of soil sampling was 30) to put into a plastic container. The soil was dissolved by 25 ml soil sample preparation solution (HANNA HI7051, Hanna Instruments Ltd, Italy). The pH meter was used to detect the soil suspension. From these recordings, calibration curve was calculated and data corrected.

Elevation was measured with GPS handheld PC (TRIMBLE GeoExplorer 3000 GeoXM Handheld, Trimble Navigation Ltd, USA). The accuracy of detecting elevation is 10 cm with an external antenna (Trimble Zephyr 2, Trimble Navigation Ltd, USA). This antenna offers precise positioning with sub-millimeter phase center accuracy and a robust low-elevation satellite tracking. Before the collection of elevation, the level of signals was at least six satellites. The elevation and geographic coordinate system information was automatically written into the meter after initiating the recording and keeping the device still for 15 seconds. The data was then exported into a computer to generate a map, using ArcGIS 10.0 (Environmental Systems Research Institute, Inc., Redlands, CA).

### Statistical analysis

Using GeoDa 1.4.0 (Spatial Analysis Laboratory, University of Illinois, Urbana-Champaign, IL, USA, https://geodacenter.asu.edu/), we first calculated the univariate LISA indicator of snails to produce a LISA cluster map of the bottomland of Dongting Lake Region. In the procedure of calculation, we chose queen contiguity as the contiguity weight.

Spatial clustering of the snails was checked. The data yielded spatial clusters (positive autocorrelation) falling into two categories (High-High and Low-Low) and two classes of outliers (negative autocorrelation, High-Low and Low-High). Inference for all Moran’s I statistics is based on permutation testing, where a reference distribution is calculated for spatial randomness and compared with the observed data over multiple iterations [43].

In the calculation of LISA, p values <0.05 were considered statistically significant. This was the unified test criterion in the following computations. The theory of this statistic is as follows:

### Local Indicators of Spatial Association, LISA

The local autocorrelation statistics for each observation is defined as the following form: [29]

$$I_{i}=Z_{i}\sum_{j}W_{\mathrm{ij}}Z_{j}$$

 *Z*_*i*_ and *Z*_*j*_ are deviations from the mean, which are standardized z-scores for each variable i and j, respectively. The standardized Z-score for each variable is computed as the observed value (e.g., water content) at location i minus the mean rate for the neighbors j (e.g., average water content) divided by the standard deviation. The summation over *j* is such that only the neighboring values *j*∈*J* are included. In this research, *I*_*i*_ denotes the LISA index of per variable in point *i* and *W*_*ij*_ is the distance-based spatial weight matrix revealing the proximity of point *i* to point *j*.

The clustering degree rises as LISA value increases. An area with high LISA value is a clustered area that should be noticed. There are four association patterns, including high-high, high-low, low-high and low-low. High-high and low-low patterns reveal that the value in one point is similar with that around it. High-low and low-high illustrates that value in one point has different neighboring values.

Second, we calculated univariate statistics and Moran’s I statistic of each variable to check distribution and to measure spatial autocorrelation. Adopting GeoDa 1.4.0. Positive values of Moran’s I statistic reveals that neighbouring points have similar values and vice versa. Spatial models are suitable for variables with significant spatial autocorrelation.

Third, since dependent value and mean snail density was not gaussian distributed, we used square root method to transform it. An ordinary least squares (OLS) regression model was fitted to estimate the association of environmental factors and mean snail density. This step was conducted in IBM SPSS 19.0 (IBM Corporation, USA). Colinearity was measured by computing variance inflation factor (VIF). Colinearity could be neglected if VIF is less than 10.

Owing to the existence of spatial autocorrelation, geographically weighted regression (GWR) more accurately analyzed the association between environmental factors and mean snail density. We used GWR 4.0 (Professor Tomoki Nakaya, The Department of Geography, Ritsumeikan University, Kyoto, Japan, http://www.st-andrews.ac.uk/geoinformatics/) to analyze the association. In the process of calculation, gaussian model and fixed gaussian kernel type were adopted. We chose an appropriate spatial weighting function based on AIC. The linear model and GWR model were compared. If the difference of AIC in these two models was more than three, GWR would be utilized as the better model, even considering the complicacy of GWR. The theory is as follows:

### Geographically weighted regression, GWR

*Y*_*i*_ denotes the dependent variable, *K* represents the number of variables, *i* is the number of samples;  *α*_0_ is a primary parameter, *ϵ*_*i*_ is the spatial error in point *i,* and the corresponding coefficient estimate is = *(X*^*T*^*X)*^*−1*^*X*^*T*^*Y*. The function is as following:

$$Y_{i}=\alpha_{0}\left(u_{i},v_{i}\right)+\sum \alpha_{k}\left(u_{i},v_{i}\right)k_{\mathrm{ik}}+\epsilon_{i}$$

 (*u*_*i*_, *v*_*i*_) is the coordinate of central point. In this study, *Y*_*i*_ is the mean snail density in point *i; k*_*ik*_ is k^th^ environmental factor in point *i*, where the subscript *k* denotes the count of environmental elements; and *ϵ*_*i*_ is the residual error. *α*_*k*_(*u*_*i*_, *v*_*i*_) represents the value of continuous function *α*_*k*_(*u*, *v*) in point *i*. In GWR [44], regression coefficient is not a global unified value, but a parameter that will change in different locations. These estimates based on geographical space describe how the parameter changes with the variation of space. Therefore, we can explore the spatial heterogeneity in these variables.

Fourth, values in the points possessing spatial clustering were extracted. Univariate statistics were displayed according to two groups (high-high and low-low). Similarly, we extracted significant points in the GWR model and analyzed univariate statistics in two groups (positive correlation and negative association).

Finally, interquartile ranges in these two tables of environmental factors were compared with the reported reference scopes suitable for snail habitation and reproduction.

## Results

### Univariate analyses

In this study, a total of 8,589 snail specimens were collected from 397 sampling sites. The range of snail density was 0 to 148 snails per 0.11 m^2^, and the mean value was 23.58 with a standard deviation of 24.82. After square root transformation, the range changed to be 0 to 12.17, and the mean value became 5.79 with a standard deviation of 2.99. Therefore, the transformation made the dependent variable approach gaussian distribution.

The range of humidity was from 0.07% to 0.99%, and the mean value of it was 0.67% with the standard deviation of 0.26%; the average value of elevation was 27.97 m with the standard deviation of 6.11 m as its range was from 18.45 to 52.67 m. The overall range of pH was from 5.23 to 8.70 with a mean value of 6.77 and the standard deviation of 0.37; the average value of temperature was 24.36°C with its range from 20.20 to 32.10°C and the STD of 1.80°C.

Both the mean snail density and its transformed value had high spatial autocorrelation (both Moran’s I values were 0.50), demonstrating that sampling points with high snail density tended to be located close to other similar high value points.

Besides the mean snail density, three environmental factors including water content, pH and temperature had high spatial autocorrelation. Elevation was much lower, but it was also spatially autocorrelated.

### Spatial distribution of snails

**Figure 2:**
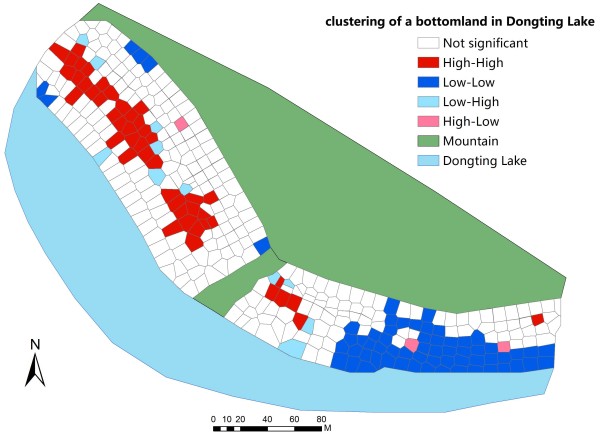
**LISA cluster of mean snail density in a bottomland of Dongting Lake Region.** Detailed legends: This map shows the spatial cluster locations.

Figure 2 shows the local spatial pattern of mean snail density. High-high and low-low locations were typically spatially clustered, while high-low and low-high locations are spatial outliers. It was worth noticing that LISA cluster map only refers to the core of the cluster. The cluster was classified as such when the value at a location is more similar to its neighbors than would be the case under spatial randomness. The clustering of high-high mean snail density was located in the middle region of the bottomland study area, while the clustering of low-low mean snail density was mainly situated at the margin of the bottomland (Figure 1).

### Multivariate analyses

Table 1 presents the results of the full regression model. Since all the variance inflation factors of independent variables were below 10, multicollinearity was not problematic. Among four parameters, water content and pH were statistically significant, showing a negative association with mean snail density. Elevation and temperature had no statistical significance.

**Table 1:** OLS regression model: association between mean snail density and natural characteristics in Dongting Lake Region

**Model Fit**						
R^2^	Adjusted R^2^		Log likelihood		AIC	
0.14	0.13		−866.91		1743.82	
**Model Estimation**						
	VIF	β	SD	t	P	
Constant		17.04	3.38	5.05	0.00	**
Water- content (%)	1.46	−0.03	0.01	−3.78	0.00	**
Elevation (m)	1.16	0.02	0.03	0.75	0.45	*
PH	1.45	−1.30	0.48	−2.72	0.01	**
Temperature (°C)	1.26	−0.13	0.09	−1.41	0.16	*
**Diagnostic Tests**						
	Tests		DF		Value	P
Normality of errors	Jarque-Bera		2		0.03	0.99
Heteroskedasticity	Breusch-Pagan		4		12.97	0.01
	Koenker-Bassett		4		21.06	0.00
	White		14		54.99	0.00
Spatial dependence	Moran’sI(error)		0.48		14.79	0.00

**P < 0.05, *P > 0.05.

The fit of the OLS model was not good (Log likelihood =-8, AIC = 1743.82, Adjusted R^2^ = 0.13). The high probabilities of the Jarque-Bera score indicated gaussian distribution of the error. Low probabilities of White test, Breusch-Pagan and Koenker-Bassett scores showed the existence of heteroskedasticity (P < 0.05). Moran’s I (error) score was positive and highly significant (P < 0.05), indicating a strong positive spatial autocorrelation of the residuals.

As the dependent variable and independent variables were spatially autocorrelated and locally distributed, we used geographically weighted regression (GWR) to analyze the association between them. In the GWR model, every sampling point would produce a specified model containing coefficients and its p-value. Compared with OLS model, the regression coefficients changed considerably. Environmental factors had both negative association and positive association with the square root of mean snail density, as different sampling points had varying local conditions. At the same time, the significance of the four covariates also changed.

Table 2 reveals the results of the GWR model. Contrasting the model fit part in OLS and GWR models, it was certain that GWR performed better. In the diagnostic assessment of GWR, the results demonstrated that GWR was better than global spatial regression models. The low possibility of GWR ANOVA test reported that GWR had some improvements. The significant F statistic resulting from the ANOVA test was utilized to establish whether the GWR model provides a better fit over the global regression model, and these values support the use of GWR. The DIFF of Criterion was the result of a test of spatial variability in a variable’s coefficient (based on an AIC criterion). DIFF of four independent variables had negative values, indicating that GWR was better than global analysis. DOF was the degree of freedom in the ANOVA analysis.Collectively, GWR was established as a good performing model for these variables.

**Table 2:** GWR models: association between mean snail density and natural characteristics in Dongting Lake region

**Model Fit**								
R^2^	Adjusted R^2^			Log likelihood			AIC	
0.74	0.65			1305.35			1446.73	
**Model Estimation**								
	β				t			
	Min	Max	Mean	STD	Min	Max	Mean	STD
Constant	2.14	7.28	5.11	1.32	2.80	19.60	11.65	3.64
Water- content(%)	−5.45	3.18	−0.61	2.10	−8.07	6.44	−0.94	4.30
Elevation(m)	−1.89	1.16	−0.02	0.49	−4.23	2.62	0.02	1.22
PH	−2.02	0.86	−0.17	0.59	−3.55	2.45	−0.20	1.17
Temperature(°C)	−1.52	0.72	−0.10	0.42	−3.86	2.10	−0.21	0.99
**Diagnostic Tests**								
GWR Anova Table								
	SS		DF	MS		F		P
Global Residuals	2717.14		5.00					
GWR Improvement	1901.67		88.27	21.54				
GWR Residuals	815.48		262.73	3.10		6.94		<0.05
Geographical variability tests of local coefficients								
	F			DOF for F test			DIFF of Criterion	
Constant	4.19			2.83			−8.61	
Water content	7.39			3.20			−25.92	
Elevation	5.77			3.28			−12.03	
PH	6.84			3.31			−15.74	
Temperature	279.51			3.07			−388.96	

**Figure 3:**
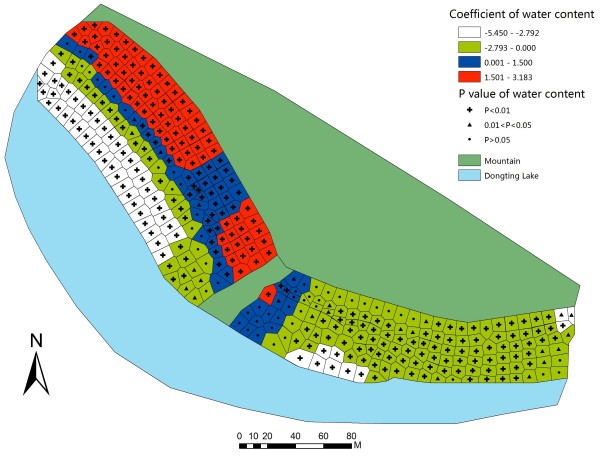
**Coefficient and significance of water content in GWR model.** Detailed legends: This map shows the value of parameters and its significance in GWR model to illustrate how water content influences mean snail density.

**Figure 4:**
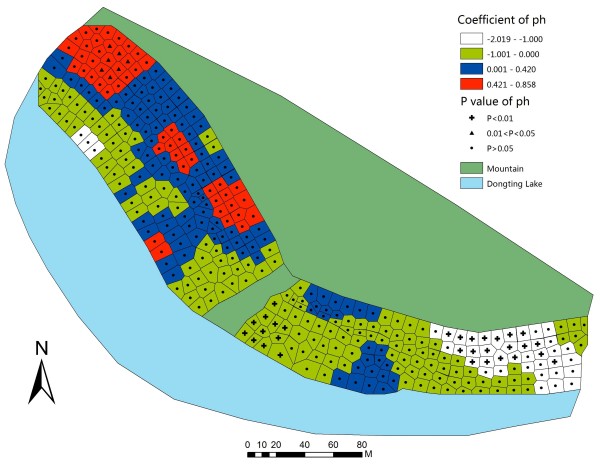
**Coefficient and significance of pH in GWR model.** Detailed legends: This map shows the value of parameters and its significance in GWR model to illustrate how pH influences mean snail density.

**Figure 5:**
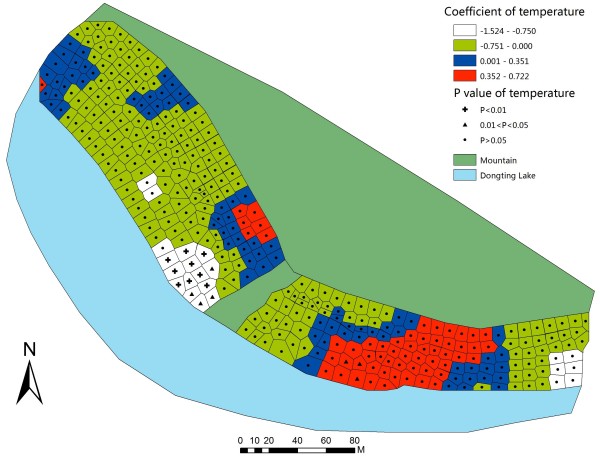
**Coefficient and significance of temperature in GWR model.** Detailed legends: This map shows the value of parameters and its significance in GWR model to illustrate how temperature influences mean snail density.

**Figure 6:**
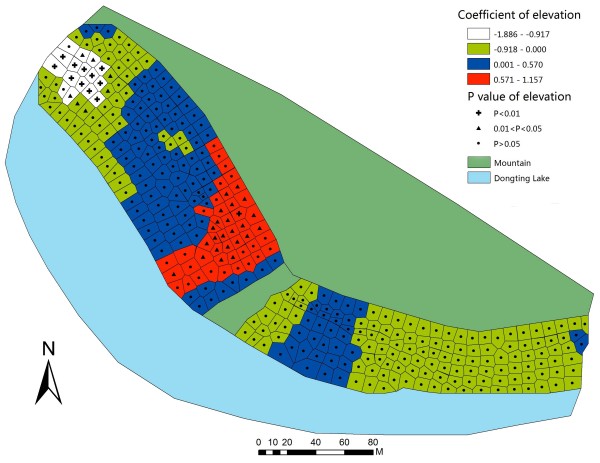
**Coefficient and significance of elevation in GWR model.** Detailed legends: This map shows the value of parameters and its significance in GWR model to illustrate how elevation influences mean snail density.

To further illustrate the coefficients and the relevant P-value in GWR model, GWR parameter maps of each covariate were constructed. In the northwestern part of the studied bottomland, the GWR map of water content (Figure 3) indicated that, the mean snail density was positively associated with water content when the range of the coefficient was from 1.50 to 3.78. The map illustrated that an increase of water content might generate an increase in mean snail density in a certain area. Meanwhile, it suggested that the relationship between water content and snails was positive but not linear. Three other factors had statistically significant positive associations with mean snail density in the areas. The ranges of coefficients were from 0.42 to 0.86 (pH, Figure 4), from 0.35 to 0.72 (temperature, Figure 5), and from 0.57 to 1.16 (elevation, Figure 6).

### Suitable range analyses

According to the results of the LISA cluster analysis, snail habitats were divided into high-high clustered areas and low-low clustered areas. Therefore, as illustrated in Table 3, four environmental factors were divided into high-high and low-low groups respectively. Since all the factors did not follow gaussian distribution, inter-quartile range was a better parameter to illustrate a hospitable range for snail survival.

**Table 3:** Univariate statistics of the independent variables of four clusters in Dongting Lake Region

		**N**	**Median**	**Percentiles**	**Percentiles**
				**(25%)**	**(75%)**
Water content (%)	High-High	44	65.90	58.70	68.93
	Low-Low	54	99.90	99.90	99.90
Elevation(m)	High-High	44	26.66	23.50	30.39
	Low-Low	54	27.87	25.97	34.60
PH	High-High	44	6.65	6.56	6.85
	Low-Low	54	7.05	6.93	7.20
Temperature(°C)	High-High	44	23.50	22.73	24.28
	Low-Low	54	25.40	25.10	25.83

The inter-quartile range of high-high clustered area in water content was from 58.70% to 58.93%, suggesting that snails would survive and reproduce largely in that region. Similarly, the interquartile range of the low-low clustered area was from 70.00% to 99.90%. The low-low clustered area indicated that snails would not thrive in this area.

For inter-quartile range of elevation, the scope of high-high clustered area was from 23.50 m to 30.39 m, while the range of low-low clustered area was from 25.97 m to 34.60 m. For inter-quartile range of pH, the range of high-high clustered area was from 6.56 to 6.85, as the scope of low-low clustered area was from 6.93 to 7.20. For temperature, the range of high-high clustered area was from 22.73°C to 24.28°C, while the scope of low-low clustered area was from 25.10°C to 25.83°C.

Table 4 presents that water content is positively associated with mean snail density when the water content is between 36.58% and 61.08%. However, this relationship was negatively associated when within the range of 66.60% to 99.90%. Similarly, elevation and mean snail density had positive correlation when elevation range was from 24.79 m to 29.44 m, and they had negative correlation when elevation range was from 21.85 m to 25.25 m. PH and mean snail density were positively correlated in pH range from 6.54 to 6.89, but were negatively correlated in pH range from 6.73 to 7.05. Temperature and mean snail density had positive association as temperature scope was from 24.30°C to 25.70°C, while the negative scope was from 22.40°C to 24.15°C.

**Table 4:** Univariate statistics of the independent variables of linear correlation in GWR in Dongting Lake Region

		**N**	**Median**	**Percentiles**	**Percentiles**
				**(25%)**	**(75%)**
Water content (%)	positive	118	52.30	36.58	61.08
	negative	185	79.20	66.60	99.90
Elevation(m)	positive	24	27.60	24.79	29.44
	negative	18	24.12	21.85	25.25
PH	positive	7	6.69	6.54	6.89
	negative	30	6.93	6.73	7.05
Temperature(°C)	positive	4	25.00	24.30	25.70
	negative	13	23.60	22.40	24.15

## Discussion

*Oncomelania hupensis* is the unique intermediate host of *S. japonica* in China, and its distribution is highly consistent with the *S. japonica* epidemic area [45]. The survival, reproduction and spread of these snails are often influenced by many factors, and among these elements, environmental factors (i.e. climate, hydrology, vegetation, and sunlight) play a significant role [46]. In many past studies, the effects of these factors on snails have alone been analyzed in a laboratory setting [46-50] or a field [51,52]. However, these factors have interactions so that occasional synergetic effects can be generated [53]. Hence, we detected the four main environmental factors (i.e. water content, pH, temperature of soil and elevation) in the bottomland of Dongting Lake Region, and included these four elements, into the spatial model to analyze the complex collective effects of the elements on snails. Our results showed that snails had obvious spatial clustering areas in the bottomland (high-high clustering areas are located in the northwestern bottomland, while low-low clustering areas are located in the southeastern bottomland). This finding of the spatial cluster of snails was consistent with previous reports [54,55]. Based on this finding, the ranges of the environmental factors in these high-high clustered areas of snails might be the suitable ranges for these snails, while the scopes of circumstance elements in the low-low clustered areas possibly generate unsuitable ranges for snails. According to this hypothesis, we discussed the possible optimum scopes. Meanwhile, we used the GWR model to analyze the association between snails and environmental factors, so we could provide an alternative means of identifying the possible optimum ranges for the snails.

The results of this study showed that the range of 58.70% to 68.93% was the moisture content scope in the high-high clustered areas of snails, while in the low-low clustered areas, most of the water content values were 99.90%, with some values being below 30.00%. Therefore, snails might not survive when water content of soil is more than 99.90% or less than 30.00%. This indicated that mean snail density and moisture content had a U-shaped association, thus snails would live and breed in this given water range. Snails might start to migrate out of their habitat if water content was out of this suitable scope. Chen *et al.*[56] reported that a small number of snails began to move when water content was more than 12.00%, about one fifth snails would start moving at 20.00% water content, about half of snails would move at 30.00% water content, and the snails would be very active at 40.00% water content. However, when water content was more than 80.00%, mean snail density would decline [56]. Similarly, the association in GWR model showed that the mean snail density increased gradually when the water scope was between 36.58 and 61.08%, while they decreased gradually as the range was from 66.60 to 99.90%. These further supported our previous results. Considering the outcomes of past and current experiments, we supposed that 58.70% to 68.93% might be a suitable range of water moisture in soil for the snails in our study site.

In the high-high clustered areas of snails, the elevation was from 23.50 m to 30.39 m, while in the low-low clustered regions, the elevation was from 25.97 m to 34.60 m. These two kinds of clustered ranges of elevation had some interactions, and it might be related to only the elevation considered and other factors may not be involved. When the four factors were analyzed together in the GWR model, our results showed that the mean snail density increased gradually when the elevation was from 24.79 m to 29.44 m, while the mean snail density decreased gradually when the elevation was from 25.25 m to 21.85 m. Previously reported ranges from studies being conducted in eastern Dongting Lake Region include optimal elevations of 24.50 m to 26.00 m in Dongkou [51] and 25.00 m to 27.50 m in Matang [52]. Based on the results of the previous literature and this study, 23.5 m to 26.0 m might be an optimum scope of the elevation for snails in our study field.

The results of pH showed that high-high clustered range was from 6.56 to 6.85, and low-low clustered range was between 6.93 and 7.20. The ranges of high-high clustered and low-low clustered were both in the range 6.7 to 7.8, as reported in Table 5. The results of GWR model highlighted that the mean snail density increased gradually when the pH was between 6.54 and 6.89, while mean snail density decreased gradually when the pH was from 6.73 to 7.05. Based on the results of the previous reports and this study, 6.6 to 7.0 might be a suitable range of pH for snails in this study field.

**Table 5:** Reported ranges of four environmental factors suitable for snail existence

**Author/date**	**Method**	**Referenc range**			
		**Water-content(%)**	**Temperature(°C)**	**PH**	**Elevaton(m)**
OuYang *et al.* 2009 [57]	Survey	-	13 ~ 25	-	-
Xu *et al.* 2001 [49]	Experiment	40	20~30	-	-
Wang *et al*. 2007 [47]	Experiment	-	20 ~ 30	-	-
Hu *et al*. 2010 [52]	Survey	-	-	-	25.0 ~ 27.5
Lu *et al*. 2013 [48]	Experiment	-	20~25	-	-
Luo *et al.* 2012 [51]	Survey	-	-	-	24.5~26.0
Yang *et al*. 2009 [46]	Experiment	30	20~26	6.7 ~ 7.8	-
Zhou *et al.* 2005 [50]	Experiment	-	20~25	6.8 ~ 7.5	-

The high-high clustered range of temperature was from 22.73°C to 24.28°C, as the low-low clustered scope of that was from 25.10°C to 25.83°C. Su *et al*. (1963) reported that snail survival would be threatened if the temperature was less than 5.00°C or more than 35.00°C, and the snail would not eat food past those parameters [58]. The results of the GWR model presented show that the mean snail density increased gradually when the temperature was between 24.30°C and 25.70°C, while mean snail density decreased gradually when the temperature was from 24.15°C to 22.40°C. Considering the results in this study and previous studies, a possible suitable range of temperature was from 22.73°C to 24.23°C in our study site. It is worth noting that uncertainty existed in the process of model fitting.

The optimum temperature range in this study presented a narrower range in comparison to prior literature. This difference might be attributed to a new method applied during our study. In contrast to previous research, information of environmental factors was collected in our study sites, whereas data of environmental elements were gathered in laboratories among previous studies. The data was then analyzed by local indicators of spatial autocorrelation and spatial regression model, which took the spatial attributes into account. In previous literature, descriptive methods and linear models were commonly adopted. However, some limitations may exist with these past approaches. First, experimental data might not reflect the real situation in the sampling sites, since the delivery of samples and microenvironment in a laboratory might change the content in the samples. Second, descriptive methods and linear model did not consider the spatial clustering in snails in the process of analysis.

Several limitations were highlighted in our study. First, the sampling of snails and environmental elements were conducted only in summer. Further studies could be carried out during spring in the same positions. Second, our study was mainly carried out in bottomland and water. The sampling sites could be expanded in future research.

## Conclusion

In summary, this research conducted a field survey, collected data in the sampling sites and analyzed the data using spatial techniques. Compared with previous reports, the explorations in this study adopted field research instead of experimental and used a new method to analyze data. Natural factors of the soil might decide snail survival, and the relationship between mean snail density and natural factors (e.g., moisture content) was nonlinear. Indeed, it follows a U-shaped curve as the snails do not seem to survive when the soil water content is either extremely high (99.9%) or less than 30%, leading to snail dispersion outside the optimal range of 59%-69%. These findings might contribute to better prediction and control of snail habitat dynamics, leading to more accurate prevention of *S. japonica* transmission.

## Competing interests

All authors declare that they have no competing interests.

## Authors’ contributions

YZ, GR and QJ conceived the study; JW, YZ, GR, LL, SZ, XS, ZH, BC, JY, and QJ performed the field collections; JW, SL, AC and YZ wrote the manuscript; JW performed statistical analyses. All the authors read and approved the final version of the manuscript.
